# Physiological Changes and Trimester-Specific Reference Intervals for Complete Blood Count Parameters in Korean Pregnant Women

**DOI:** 10.3390/medicina61091665

**Published:** 2025-09-14

**Authors:** Heejin So, Kyungsuk Kwon, Sukhyun Jung, Kyeongmi Kim

**Affiliations:** 1 Department of Laboratory Medicine, Gimpo Woori Hospital, Gimpo 10099, Republic of Korea; 2 Department of Laboratory Medicine, CHA Ilsan Medical Center, CHA University School of Medicine, Goyang 10414, Republic of Korea

**Keywords:** complete blood cell, pregnant women, trimester, reference interval

## Abstract

*Background and Objectives*: Pregnancy induces numerous physiological changes, including hematologic adaptations, which affect complete blood count (CBC) parameters. Existing reference intervals for CBC are often based on non-pregnant populations, potentially limiting their clinical utility during pregnancy. This study aimed to evaluate longitudinal changes in CBC parameters throughout pregnancy in Korean women and to establish gestational age-specific reference intervals. *Materials and Methods*: This retrospective study, conducted between March and May 2025, included CBC tests consecutively performed on the same individuals at five time points: the first trimester (≤12 weeks), second trimester (13–28 weeks), third trimester (29–40 weeks), delivery day, and the second postpartum day. Additionally, to prevent duplication with the primary cohort, CBC data from pregnant outpatients and non-pregnant controls were also analyzed to establish reference intervals. CBC parameters were measured using an automated hematology analyzer. Reference intervals were established using the 2.5th and 97.5th percentile of the distribution. *Results*: During pregnancy, white blood cell (WBC) counts increased most significantly during the second trimester, while hemoglobin (Hgb) levels declined most markedly at this stage. Platelet (PLT) counts showed a consistent and progressive decline. The reference intervals for CBC parameters—WBC (×10^9^/L), Hgb (g/dL), and PLT (×10^9^/L)—were 5.11–12.14, 11.3–14.3, and 184–374 in the first trimester; 6.11–13.45, 10.1–13.3, and 164–356 in the second trimester; and 5.62–12.42, 10.1–14.1, and 145–349 in the third trimester, respectively. *Conclusions*: This study examined longitudinal changes in CBC parameters in Korean pregnant women and provided gestational age-specific reference intervals for CBC. This is expected to help clinicians interpret CBC results in pregnant women.

## 1. Introduction

During normal pregnancy status, the mother undergoes numerous physical and physiological changes to support fetal growth and development [1,2]. These changes affect nearly all organ systems and are mostly reversed following childbirth, returning the maternal body to its non-pregnant state [2]. Therefore, understanding the physiological changes that occur during pregnancy is critical for differentiating normal maternal adaptations from pathological or abnormal conditions.

Complete blood count (CBC) is one of the most commonly performed laboratory tests and is routinely used during pregnancy to aid in the diagnosis of anemia, infection, and other hematologic abnormalities. Although CBC testing has limitations as a screening tool for detecting abnormalities in asymptomatic individuals of the general population [3,4], it provides valuable information for assessing the physiological status of pregnant women [5]. However, despite numerous studies on hematologic changes during pregnancy [6,7,8], most clinical laboratories still provide reference values based on healthy non-pregnant adults, except for a few parameters. As a result, these ranges may fail to adequately reflect the physiological changes that occur in pregnant women. Although reference intervals for CBC parameters during pregnancy have been reported in various countries, including China and Vietnam [9,10], these reference values may be affected by population-specific factors such as nutrition, ethnicity, and environmental conditions. Therefore, the direct application of these reference intervals to the Korean population may be inappropriate.

Accordingly, this study aims to evaluate longitudinal CBC changes and to establish gestational age-specific reference intervals for CBC parameters in Korean pregnant women.

## 2. Materials and Methods

### 2.1. Study Population

This retrospective study included pregnant women who were admitted for delivery at a single hospital between March and May 2025. To assess longitudinal changes during pregnancy, only 120 patients with singleton pregnancies who underwent routine CBC testing during each of the following time points were included: the first trimester (≤12 weeks of gestation), the second trimester (13–28 weeks), the third trimester (29–40 weeks), the day of delivery, and the second postpartum day. The data were obtained by reviewing electronic medical records.

Additionally, to prevent duplication with the primary cohort, a retrospective review was performed on 1039 Korean pregnant women, aged 20 to 45 years, who visited the outpatient clinic during the same study period and underwent routine CBC testing. Based on gestational age at the time of testing, subjects were categorized into first, second, or third trimester. Only pregnant women with singleton pregnancies were included. Pregnant women with pregnancy-related complications such as hypertension, diabetes, or cardiovascular disease, as well as those with a history of visits to other medical departments for underlying conditions unrelated to obstetric care, were excluded from the study. Specifically, patients who had received iron supplementation, blood transfusions, or other hematological treatments were excluded. Women aged 20 to 45 years who visited the hospital for routine health check-ups during the study period were included as the control group. There were no significant differences in age, body mass index, or timing of the tests compared to the pregnant group.

### 2.2. CBC Results

CBC counts were measured using an automated hematology analyzer (DxH-800; Beckman Coulter, Pasadena, CA, USA). Data were collected for the following CBC parameters: white blood cells (WBC), red blood cells (RBC), hemoglobin (Hgb), hematocrit (Hct), mean corpuscular volume (MCV), mean corpuscular hemoglobin (MCH), mean corpuscular hemoglobin concentration (MCHC), red blood cell distribution width (RDW), platelet (PLT), plateletcrit (PCT), mean platelet volume (MPV), platelet distribution width (PDW). The hematology analyzer used in this study was regularly calibrated and maintained according to the manufacturer’s instructions. Daily quality control procedures were conducted using three levels of control materials to verify the precision of the measurements.

### 2.3. Establishment of Reference Ranges

Reference intervals were established for each gestational period using a non-parametric method according to the clinical and laboratory standards institute (CLSI) EP28-A3c guidelines [11]. Outliers were identified and excluded using Dixon’s D/R ratio test. D is the absolute difference between the largest and the second largest value, or between the smallest and the second smallest value, and R is the range of all observations, calculated as the maximum value minus the minimum value. If D is equal to or greater than one-third of R, the corresponding observation is considered an outlier and is removed. After removing outliers, the test is repeated on the remaining data, and this process continues until no further outliers are detected. Reference limits were defined as the 2.5th and 97.5th percentiles based on a non-parametric method, and 90% confidence intervals (CIs) for these limits were also calculated.

### 2.4. Statistics

The Wilcoxon signed-rank test was used to compare longitudinal measurements during pregnancy within the same patients. Comparisons between groups were performed using the Mann–Whitney U test. A *p*-value of less than 0.05 was considered statistically significant. Statistical analyses were performed using SPSS 26 software (IBM Corp., Armonk, NY, USA).

## 3. Results

### 3.1. Longitudinal Changes Within Individual Patients

We retrospectively analyzed 120 pregnant women with singleton deliveries who had received prenatal care at our hospital starting in early pregnancy. CBC test results were collected from pregnant women aged between 27 and 45 years. During delivery, the amount of blood loss varied widely, with a minimum recorded volume of 100 mL and a maximum reaching up to 1200 mL. It was observed that pregnant women who underwent cesarean section experienced significantly greater blood loss compared to those who had vaginal deliveries, and this difference was statistically significant. Additionally, the weight of the expelled placenta among these pregnant women ranged between 425 g and 1303 g. The birth weights of the newborns also showed variation, ranging from 2070 g to 3870 g. The baseline characteristics of the 120 pregnant women are presented in Table 1.

**Figure 1:**
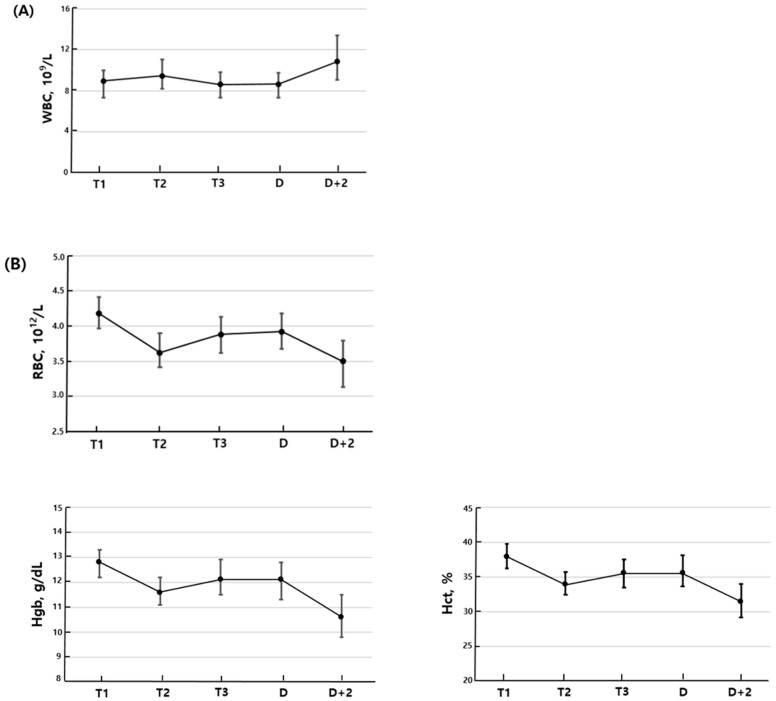

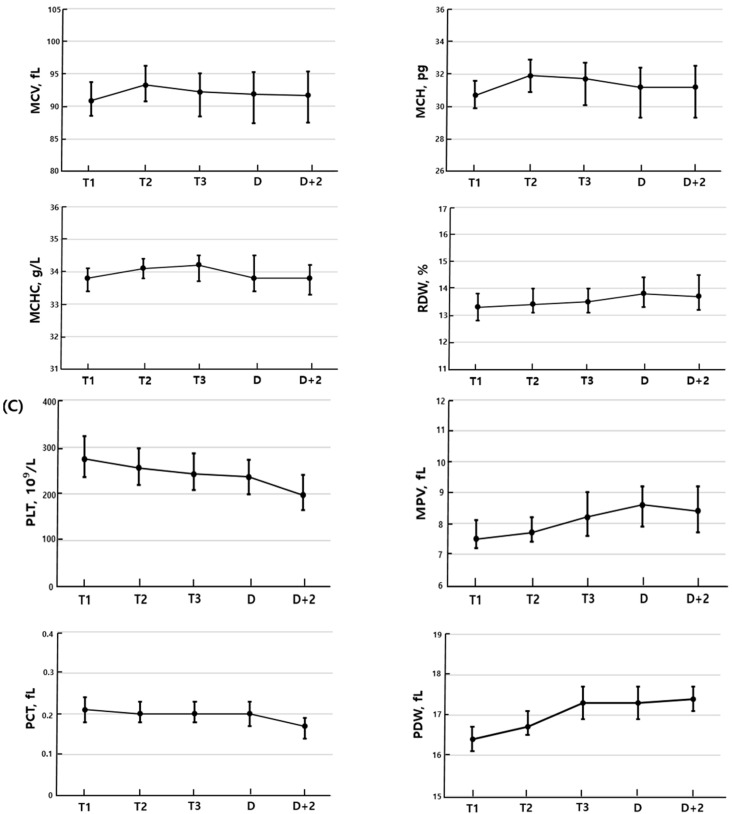
Complete blood cell parameters during pregnancy. (**A**) White blood cell, (**B**) red blood cell related parameters, (**C**) platelet related parameters. The dot indicated the median, and the bars denote the interquartile range, defined by the 25th and 75th percentiles. T1: first trimester; T2: second trimester; T3: third trimester; D: delivery day; D + 2: postpartum day 2.

Compared to the first trimester, the second trimester showed statistically significant differences in all measured parameters except for RDW. WBC, MCV, MCH, MCHC, MPV, and PDW increased, while RBC, Hgb, Hct, PLT, and PCT decreased. In the third trimester, significant changes were observed in all parameters compared to the second trimester, except for MCHC, RDW, and PCT. RBC, Hgb, Hct, MPV, and PDW increased, whereas WBC, MCV, MCH, and PLT decreased. CBC parameters including WBC, Hgb, Hct, PCT, and PDW did not differ significantly between the third trimester and the day of delivery. However, compared to the third trimester, RBC, RDW, and MPV increased on the day of delivery, while MCV, MCH, MCHC, and PLT decreased. On the second postpartum day, WBC increased compared to the day of delivery, while RBC, MCV, Hgb, Hct, PLT, and PCT decreased. There were no significant differences in MCV, MCH, MCHC, RDW, and PDW (Figure 1).

### 3.2. Reference Interval

A total of 222 individuals were included in the control group, along with 160 women in the first trimester, 382 in the second trimester, and 497 in the third trimester. The mean and median gestational ages at the first, second, and third trimesters were as follows: 10.0 ± 2.2 weeks and 10 + 2 weeks, 25.7 ± 1.4 weeks and 25 + 5 weeks, and 36.0 ± 1.7 weeks and 36 + 1 weeks, respectively. Table 2 presents a comparison of the median values and ranges of CBC parameters between the control group and pregnant women categorized by gestational age.

Additionally, Table 3 shows the reference intervals for each gestational period, established according to CLSI EP28-A3c guidelines, with the lower and upper limits defined as the 2.5th and 97.5th percentiles, respectively.

## 4. Discussion

Blood volume increases by approximately 1500–1600 mL (40–50%) during pregnancy [12,13]. This expansion is primarily due to a disproportionate increase in plasma volume relative RBC mass, resulting in a physiological hemodilution effect. As a result, pregnant women tend to have decreased Hgb and PLT counts compared to non-pregnant women [7]. WBC count may increase slightly during pregnancy as a result of physiological stress induced by the pregnant condition [8,14]. Consequently, the application of reference values derived from healthy non-pregnant adults may not be appropriate for pregnant women. For example, if an Hgb level of 10.7 g/dL is observed during the second trimester and interpreted using non-pregnant reference intervals, it could be classified as anemia, potentially leading to unnecessary iron supplementation or further investigation. Clinicians can better distinguish between physiological adaptation and true pathological conditions by using baseline intervals for each trimester of pregnancy. The present study aimed to examine the gestational age-related changes in CBC parameters and to establish pregnancy-specific reference intervals.

Pregnancy induces changes in hormone levels, such as estrogen and progesterone, which stimulate leukopoiesis in the bone marrow, leading to an increase in WBC count. Elevated levels of the stress hormone cortisol during pregnancy may contribute to leukocytosis as part of the physiological stress response. Also, leukocytosis during pregnancy is also thought to result from selective immune tolerance, immunosuppression, and immunomodulation associated with fetal development [14,15,16]. In our study, the median WBC count in the control group was 5.45 × 10^9^/L, whereas in pregnant women it increased to 8.86 × 10^9^/L in the first trimester, 9.39 × 10^9^/L in the second trimester, and 8.53 × 10^9^/L in the third trimester. These results were consistent with those reported in previous studies [9,10]. The observed increase in WBC count during pregnancy is mainly due to a rise in neutrophils. This phenomenon is thought to result from a combination of factors, including the redistribution of neutrophils between the marginal pool and the circulating pool, as well as a decreased rate of neutrophil apoptosis, which prolongs their lifespan in the bloodstream [1,8,17]. Although changes in neutrophil counts during pregnancy are well established, our study did not evaluate these parameters [17]. The absence of differential WBC count data in this study is one of its limitations.

Hgb levels began to decline during the first trimester, reached their lowest point in the second trimester, and then increased again in the third trimester. This is due to an increase in maternal plasma volume starting early in pregnancy, with the greatest expansion occurring during the second trimester, resulting in a relative dilution of the blood [18]. Additionally, the increased iron demand for fetal growth and placental development may lead to maternal iron deficiency, which can impair erythropoiesis [19,20]. This is a normal part of the physiological changes during pregnancy. In fact, the World Health Organization (WHO) and the Centers for Disease Control and Prevention (CDC) define anemia as hemoglobin levels below 12 g/dL in non-pregnant adult women, whereas for pregnant women, anemia is defined as hemoglobin below 11 g/dL in the first and third trimesters, and below 10.5 g/dL in the second trimester [21,22]. In our study, the lower reference limits (2.5th percentiles) for hemoglobin in the first, second, and third trimesters were 11.3, 10.1, and 10.1 g/dL, respectively. While the first trimester value met the anemia guidelines proposed by the WHO and CDC, the values for the second and third trimesters were lower than those recommended. In studies conducted on Chinese populations, such as those by Aiwei Li et al. and Yi Jin et al., as well as in research on Vietnamese populations by Pham et al., the 2.5th percentile values were found to be below the established reference limits [9,10,23]. In studies conducted on Korean populations, 2.8, 22.5, and 27.1% of participants in the first, second, and third trimesters, respectively, were classified as anemic according to the anemia diagnostic criteria established by the WHO and CDC [24]. Since the WHO and CDC reference values are established based on populations of non-Korean ethnicities, it is more appropriate to consider anemia thresholds for Korean pregnant women not solely based on a hemoglobin cutoff of 11 g/dL, but rather in conjunction with the red blood cell parameters and gestational age-specific hemoglobin reference intervals established in this study.

During pregnancy, PLT counts decrease due to hemodilution and increased platelet consumption, which are generally considered physiological changes. Especially in late pregnancy, activation of the coagulation system leads to further increased platelet consumption [25]. For this reason, approximately 7–10% of pregnant women experience gestational thrombocytopenia [26]. In our study, platelet counts showed a progressive decline from the first to the third trimester. Using a reference value of 150 × 10^3^/µL to define normal platelet levels, thrombocytopenia was most frequently observed in the third trimester, affecting 1.8% of the participants in this study. Among these individuals, no clinical sign and symptom associated with thrombocytopenia were observed.

Serial CBC measurements in pregnant women also demonstrated findings consistent with those reported in the studies mentioned above. WBC counts increased most significantly during the second trimester, while Hgb levels declined most markedly at this stage. PLT counts showed a consistent and progressive decline beginning in early pregnancy and continuing throughout gestation. There was no statistically significant difference in WBC and Hgb levels between the third trimester and the day of delivery. However, PLT counts showed a significant difference, which is likely attributable to increased PLT consumption due to activation of the coagulation system. Gestational thrombocytopenia is known to occur more frequently in the second or third trimester rather than in the first trimester. However, if the PLT count remains above 100,000/µL without any related symptoms, no specific treatment or intervention is usually required [27]. After delivery, an increase in WBC count and decreases in Hgb and PLT counts were observed, likely due to bleeding associated with cesarean section or vaginal delivery.

Reference values are defined as numerical values established based on measurements obtained from a reference population, which is selected according to clearly defined criteria. The most commonly used type of reference value is the reference interval, which is defined by the lower and upper limits that include the central 95% of observed values in the reference group [28]. In general, reference intervals are established through statistical analysis of laboratory measurements obtained from healthy adults, and they serve as important criteria for screening specific diseases or evaluating health status in asymptomatic populations. Reference values in CBC tests can be influenced by various factors such as age, sex, ethnicity and genetic background, geographic location, diet and nutritional status, pregnancy, and other related factors [29,30]. However, in certain population groups, such as children, the elderly, and pregnant women, conventional reference intervals may not adequately reflect physiological changes associated with growth or aging, presenting a significant limitation. Consequently, the need to establish population-specific reference values has been increasingly recognized, and numerous studies have been conducted worldwide to analyze reference values for CBC parameters according to demographic characteristics [29,30,31,32].

Although studies to establish gestational age-specific reference values for CBC parameters in pregnant women have been performed in a number of countries, such research is limited in Korean women. This study presents gestational age-specific reference intervals derived from a cohort of Korean pregnant women and compares these findings with previously reported reference intervals from East Asian (Chinese), Southeast Asian (Vietnamese), and African (Ethiopian) populations (Table 4) [9,10,33]. In all four studies, WBC counts were highest during the second trimester. Hgb levels were lowest in the second trimester in all studies except for the one conducted on the Chinese population. PLT counts showed a decreasing trend across the first, second, and third trimesters in this study as well as in the studies involving Chinese and Ethiopian populations. Therefore, consistent trends in WBC, Hgb, and PLT levels according to gestational age were observed across all four studies. However, the reference intervals differed in absolute values across countries. For Hgb, the lower limits reported in the Chinese study were 8.8 g/dL and 8.4 g/dL for the second and third trimesters, respectively. PLT counts in the Chinese population were lower than those observed in other countries, with values of 148 × 10^3^/μL, 111 × 10^3^/μL, and 80 × 10^3^/μL for the first, second, and third trimesters, respectively. In the Vietnamese study, MCV and MCH were notably lower compared to those reported in other countries. One possible explanation is the high prevalence of thalassemia gene carriers in Vietnam, which is approximately 13.8%, significantly higher than the global average of around 1.5% [34]. This high carrier rate may contribute to the predominance of microcytic, hypochromic red blood cells observed in the population. PCT and PDW are known as useful indicators for assessing the risk and severity of preeclampsia [35,36]. In our study, PCT showed a decreasing trend toward the late stages of pregnancy, while PDW tended to increase. However, there were no reported results for PCT and PDW in studies involving Chinese and Vietnamese populations, and findings from Ethiopian populations differed from our study results. These differences in CBC reference values among various countries are believed to result from a complex interplay of factors, including ethnic diversity, geographic location, and nutritional status.

Asians tend to have lower WBC, Hgb, and Hct levels compared to Caucasians, while PLT counts tend to be higher [31]. Among Asians, the prevalence of thalassemia is less than 1% in Korea, which is lower than in Vietnam, where it is reported to be 13.8% [37]. Along with the influence of ethnicity and disease prevalence on CBC results, it is important to recognize that physiological changes during pregnancy, such as hemodilution and increased plasma volume, can significantly affect hematological parameters. These changes may vary in degree depending on genetic background and environmental factors, further complicating the establishment of universal reference intervals. Therefore, developing population-specific and gestational age-adjusted reference values is essential for accurate clinical assessment and management of pregnant women. This study was conducted at a single center in South Korea and included only pregnant women with singleton pregnancies. Subgroup analyses based on age, parity, or method of pregnancy were not performed. Therefore, this study may limit the generalization of the reference intervals to a wider population. Nevertheless, these reference intervals are expected to aid in the interpretation of CBC test results and clinical decision making for Korean pregnant women according to gestational age. It is expected that future multicenter or nationwide studies will enable more accurate establishment of CBC reference intervals for Korean pregnant women. Furthermore, it is expected that these CBC parameters can serve as baseline data for future studies utilizing them in conditions such as preeclampsia and depression during pregnancy [38,39].

## 5. Conclusions

This study analyzed changes in CBC parameters during gestation in Korean pregnant women and established trimester-specific reference intervals reflecting physiological changes. As a result, this study demonstrated that physiological changes during pregnancy lead to distinct differences in CBC results between pregnant and non-pregnant adults. Therefore, applying reference intervals derived from the general population for interpreting CBC results in pregnant women may lead to incorrect interpretations. The use of pregnancy-specific reference intervals is expected to help clinicians make more accurate and informed clinical decisions. This study is significant as the first to establish gestational age-specific CBC reference intervals for Korean pregnant women.

## Figures and Tables

**Table 1:** Characteristics of pregnant women with serial complete blood cell count measurements during gestation (*N* = 120).

	*N* (%)	Mean ± SD	Median
Age		35.2 ± 3.8	35
Parity			
Primipara	77 (64.2)		
Multipara	43 (35.8)		
Method of pregnancy			
Natural	71 (59.2)		
In vitro fertilization	49 (40.8)		
Delivery method			
Vaginal	31 (25.8)		
Cesarean section	89 (74.2)		
Blood loss (mL)		445.4 ± 284.6	400
Fetal weight		3131.4 ± 383.9	3160
Placenta weight		698.8 ± 153.4	670

**Table 2:** Complete blood count parameters among pregnancy trimesters and non-pregnant controls.

	Control (*N* = 222)			First Trimester (*N* = 160)				Second Trimester (*N* = 382)				Third Trimester (*N* = 497)			
Parameter	Median	Range		Median	Range		*p*1	Median	Range		*p*2	Median	Range		*p*3
		Low	High		Low	High			Low	High			Low	High	
WBC, 10^9^/L	5.45	4.00	8.66	8.86	4.57	13.56	<0.001	9.39	4.82	15.07	<0.001	8.53	4.02	13.54	<0.001
RBC, 10^12^/L	4.33	3.63	4.96	4.19	3.40	4.98	<0.001	3.61	2.93	4.33	<0.001	3.91	3.03	4.76	<0.001
Hgb, g/dL	13.3	11.5	14.9	12.9	11.2	14.5	<0.001	11.6	9.5	13.8	<0.001	12.3	9.7	14.8	<0.001
Hct, %	39.5	33.7	44.8	38.1	31.5	44.6	<0.001	33.9	27.5	40.8	<0.001	35.8	28.6	43.3	<0.001
MCV, fL	91.6	82.2	100.6	91.0	77.3	99.9	0.486	94.1	84.2	103.2	<0.001	92.8	79.5	104.1	<0.001
MCH, pg	30.9	27.0	34.3	30.8	27.3	34.3	0.567	32.3	28.3	36.1	<0.001	31.7	26.3	35.8	<0.001
MCHC, g/L	33.6	32.4	34.6	33.8	32.7	34.9	<0.001	34.2	32.9	35.6	<0.001	34.1	32.5	35.5	0.702
RDW, %	13.1	11.8	15.2	13.2	12.0	15.3	0.623	13.5	12.0	15.3	0.063	13.5	11.9	15.5	0.053
PLT, 10^9^/L	259	157	435	273	148	422	0.280	247	120	389	<0.001	235	106	372	0.005
MPV, fL	8.0	6.4	9.9	7.6	6.2	9.5	<0.001	7.8	5.9	10.1	0.007	8.3	6.1	10.9	<0.001
PCT, fL	0.21	0.13	0.34	0.21	0.13	0.32	0.175	0.20	0.11	0.29	0.002	0.20	0.12	0.27	0.374
PDW, fL	16.5	15.6	17.7	16.4	15.6	17.4	0.021	16.9	15.5	18.2	<0.001	17.3	16.1	18.7	<0.001

*p*1 values for first trimester vs. control, *p*2 values for second trimester vs. first trimester, *p*3 values for third trimester vs. second trimester.

**Table 3:** Reference intervals for complete blood cell count parameters.

Parameter	Control (*N* = 222)		First Trimester (*N* = 160)		Second Trimester (*N* = 382)		Third Trimester (*N* = 497)	
	2.5th (90% CI)	97.5th (90% CI)	2.5th (90% CI)	97.5th (90% CI)	2.5th (90% CI)	97.5th (90% CI)	2.5th (90% CI)	97.5th (90% CI)
WBC, 10^9^/L	4.06 (4.01–4.17)	8.11 (7.88–8.62)	5.11 (4.57–5.64)	12.14 (11.55–13.56)	6.11 (5.37–6.27)	13.45 (13.18–13.9)	5.62 (4.54–5.74)	12.42 (12.34–13.04)
RBC, 10^12^/L	3.76 (3.68–3.80)	4.83 (4.78–4.96)	3.69 (3.40–3.72)	4.78 (4.72–4.98)	3.09 (3.03–3.12)	4.17 (4.11–4.30)	3.26 (3.15–3.29)	4.48 (4.46–4.71)
Hgb, g/dL	11.7 (11.6–11.9)	14.6 (14.5–14.9)	11.3 (11.2–11.5)	14.3 (14.2–14.5)	10.1 (9.8–10.2)	13.3 (13.2–13.7)	10.1 (9.8–10.1)	14.1 (14.1–14.4)
Hct, %	34.3 (33.8–35.6)	43.6 (43.1–44.7)	32.8 (31.5–33.5)	43.9 (42.5–44.6)	29.2 (28.2–29.3)	39.1 (38.6–39.6)	30.2 (29.1–30.2)	41.6 (40.9–42.6)
MCV, fL	83.2 (82.4–84.3)	98.6 (97.5–99.1)	83.5 (81.0–84.2)	98.5 (97.6–99.9)	86.9 (85.4–87.7)	100.8 (100.6–101.5)	81.6 (80.9–81.8)	100.8 (100.6–102.0)
MCH, pg	27.5 (27.2–27.9)	33.2 (32.9–34.2)	28.1 (27.3–28.4)	33.9 (33.3–34.3)	28.8 (28.5–29.5)	34.9 (34.5–35.2)	26.9 (26.7–27.1)	34.9 (34.8–35.5)
MCHC, g/L	32.6 (32.5–32.7)	34.5 (34.4–34.6)	32.7 (32.7–32.9)	34.6 (34.5–34.9)	33.2 (33.1–33.3)	35.3 (35.2–35.4)	32.8 (32.7–32.8)	35.1 (35.0–35.4)
RDW, %	12.2 (12.2–12.3)	14.8 (14.6–15.2)	12.2 (12–12.3)	14.8 (14.3–15.3)	12.5 (12.4–12.6)	14.9 (14.7–15.1)	12.4 (12.3–12.4)	15.2 (15.1–15.3)
PLT, 10^9^/L	177 (158–182)	392 (367–422)	184 (148–196)	374 (370–422)	164 (156–167)	356 (345–378)	145 (121–148)	349 (348–362)
MPV, fL	6.8 (6.5–7.0)	9.7 (9.5–9.8)	6.6 (6.2–6.7)	9.1 (9.1–9.5)	6.7 (6.5–6.7)	9.5 (9.4–9.8)	6.8 (6.3–6.8)	10.3 (10.2–10.6)
PCT, fL	0.15 (0.13–0.15)	0.31 (0.3–0.33)	0.15 (0.13–0.16)	0.29 (0.27–0.32)	0.14 (0.13–0.14)	0.28 (0.27–0.29)	0.13 (0.12–0.13)	0.26 (0.26–0.27)
PDW, fL	15.8 (15.7–15.9)	17.5 (17.3–17.7)	15.7 (15.6–15.8)	17.2 (17.2–17.4)	16.1 (15.9–16.1)	18.0 (18.0–18.1)	16.4 (16.3–16.4)	18.5 (18.4–18.7)

**Table 4:** Comparison of gestational age-specific reference intervals for CBC in diverse populations.

		This Study (Korean)		Li et al. [9] (Chinese)		Pham et al. [10] (Vietnamese)		Fiseha et al. [33] (Ethiopian)	
		2.5th	97.5th	2.5th	97.5th	2.5th	97.5th	2.5th	97.5th
WBC, 10^9^/L	Trimester 1	5.11	12.14	4.68	12.87	6.33	15.24	3.6	13.2
	Trimester 2	6.11	13.45	5.97	16.78	6.99	15.55	4.56	13.59
	Trimester 3	5.62	12.42	5.53	19.56	6.22	14.14	4.56	13.62
RBC, 10^12^/L	Trimester 1	20	4.78	3.70	5.07	3.73	5.32	3.58	4.90
	Trimester 2	3.09	4.17	2.85	4.59	3.33	4.98	3.35	4.01
	Trimester 3	3.26	4.48	2.75	4.64	3.54	4.98	3.76	4.99
Hgb, g/dL	Trimester 1	11.3	14.3	11.0	14.7	10.33	13.95	10.37	13.53
	Trimester 2	10.1	13.3	8.8	13.6	9.71	13.17	9.99	12.90
	Trimester 3	10.1	14.1	8.4	14.1	9.80	13.97	10.68	13.71
Hct, %	Trimester 1	32.8	43.9	33	43	32.22	42.29	34.86	47.80
	Trimester 2	29.2	39.1	27	40	30.26	40.07	33.93	46.19
	Trimester 3	30.2	41.6	26	42	31.11	42.70	32.33	45.98
MCV, fL	Trimester 1	83.5	98.5	76.8	95.2	66.13	95.85	86.67	103.03
	Trimester 2	86.9	100.8	78.3	99.7	69.14	97.79	86.10	103.58
	Trimester 3	81.6	100.8	78.7	101.7	69.43	98.40	87.62	105.77
MCH, pg	Trimester 1	28.1	33.9	24.6	32.7	20.57	31.78	26.40	32.94
	Trimester 2	28.8	34.9	24.6	34.0	21.51	32.68	26.89	33.20
	Trimester 3	26.9	34.9	25.1	34.6	30.70	33.94	27.51	33.99
MCHC, g/L	Trimester 1	32.7	34.6	32	35.5	31.13	33.85	30.30	33.66
	Trimester 2	33.2	35.3	31.9	35.1	30.99	34.13	30.13	33.2
	Trimester 3	32.8	35.1	31.5	34.8	30.70	33.94	30.31	33.86
RDW, %	Trimester 1	12.2	14.8	11.9	16.8	12.27	17.78	12.44	15.99
	Trimester 2	12.5	14.9	12.3	17.2	12.69	16.17	12.52	17.0
	Trimester 3	12.4	15.2	12.3	19.8	12.75	17.29	12.62	16.20
PLT, 10^9^/L	Trimester 1	184	374	148	352	169.66	413.88	167.05	390.0
	Trimester 2	164	356	111	346	172.34	372.19	149.58	373.32
	Trimester 3	145	349	80	309	151.30	417.14	124.60	356.90
MPV, fL	Trimester 1	6.6	9.1	8.5	11.9	6.65	9.85	6.73	9.80
	Trimester 2	6.7	9.5	7.0	11.8	6.48	9.70	7.05	10.25
	Trimester 3	6.8	10.3	7.0	12.9	6.69	10.39	7.40	10.30
PCT, fL	Trimester 1	0.15	0.29					0.152	0.316
	Trimester 2	0.14	0.28					0.110	0.321
	Trimester 3	0.13	0.26					0.118	0.321
PDW, fL	Trimester 1	15.7	17.2	9.0	16.4			15.10	16.36
	Trimester 2	16.1	18.0	9.1	18.1			15.22	16.48
	Trimester 3	16.4	18.5	10.2	19.1			15.16	16.57

## Data Availability

Data is contained within the article.

## Abbreviations

The following abbreviations are used in this manuscript:

CBC	Complete blood count
WBC	White blood cell
RBC	Red blood cells
Hgb	Hemoglobin
MCV	Mean corpuscular volume
MCH	Mean corpuscular hemoglobin
MCHC	Mean corpuscular hemoglobin concentration
RDW	Red blood cell distribution width
PLT	Platelet
PCT	Plateletcrit
MPV	Mean platelet volume
PDW	Platelet distribution width
CLSI	Clinical and laboratory standards institute
CIs	Confidence intervals
WHO	World Health Organization
CDC	Centers for Disease Control and Prevention
